# P-939. Safety and Efficacy of Daptomycin Dose Standardization

**DOI:** 10.1093/ofid/ofaf695.1142

**Published:** 2026-01-11

**Authors:** Megan Bernabe, Sarah Dumachi

**Affiliations:** Mercy Medical Center, Cedar Rapids, IA; Mercy Medical Center, Cedar Rapids, IA

## Abstract

**Background:**

In clinical practice, daptomycin dosing frequently deviates from approved Food and Drug Administration dosing, with higher doses (≥ 8 mg/kg) increasingly recommended for the treatment of invasive infections. Daptomycin dosing policies can be implemented to improve the selection of evidence-based doses, reduce errors and waste, and streamline daptomycin preparation and administration. The purpose of this study was to assess whether standardized daptomycin dosing is as safe and effective as individualized weight-based dosing.

**Methods:**

This was a single-center retrospective cohort study of adult patients who received daptomycin for at least five consecutive days between January 2023 and December 2024. Two dosing strategies were compared: individualized weight-based dosing and standardized dosing, the latter of which involved rounding calculated doses to predefined values based on the infection type, target mg/kg dose, and patient weight range. Except for cases of endocarditis or infections involving retained hardware, adjusted body weight was used for obese patients. Outcomes assessed include the incidence of adverse effects, infection resolution, and infection recurrence within 90 days of treatment.

**Results:**

Of 162 patients identified, 86 received weight-based dosing and 76 received standardized dosing. The mean daptomycin dose was higher in the standardized group compared to the weight-based group (749 mg vs 643 mg). Other baseline characteristics were similar between groups. The incidence of creatine kinase elevation or myalgia leading to therapy modification did not differ significantly between groups. The incidence of possible daptomycin-induced eosinophilic pneumonia (DIEP) was numerically higher in the standardized dosing group (6 patients, 7.9%) versus the weight-based dosing group (3 patients, 3.5%), although not statistically significant (p = 0.22). Infection resolution and recurrence within 90 days were similar across both groups.

**Conclusion:**

Standardized dosing may be a safe and effective alternative to individualized weight-based dosing, though further evaluation of DIEP risk is warranted.

**Disclosures:**

All Authors: No reported disclosures

